# Radiographic Outcomes After Growing Rod Treatment in Early-Onset Scoliosis: With Versus Without Final Fusion Surgery

**DOI:** 10.3390/jcm14207184

**Published:** 2025-10-12

**Authors:** Yunjin Nam, Udit Patel, Sangmin Lee, Jungwook Lim, Jae Hyuk Yang, Seung Woo Suh

**Affiliations:** 1 Department of Orthopedic Surgery, Korea University Guro Hospital, Seoul 08308, Republic of Korea; nam.yunjin@gmail.com (Y.N.); uditpatel27@gmail.com (U.P.); puhoo10@naver.com (S.L.); jlim2012@gmail.com (J.L.); 2 Department of Orthopedic Surgery, Korea University Anam Hospital, Seoul 02841, Republic of Korea; kuspine@naver.com

**Keywords:** early-onset scoliosis, growing rod, final fusion, radiographic outcome, thoracic kyphosis

## Abstract

**Background/Objectives**: Early-onset scoliosis (EOS) is often treated with growing rods, which use distraction-based correction to control deformity while allowing spinal growth. Although effective in the coronal plane, this technique may adversely affect sagittal alignment, particularly thoracic kyphosis and lumbar lordosis. Whether final fusion surgery is necessary after the growing rod treatment remains controversial. This study compared radiographic outcomes, including coronal and sagittal parameters, between patients with and without final fusion to clarify the value of final fusion. **Methods**: We retrospectively reviewed 19 EOS patients treated with growing rods between 2015 and 2019. Patients undergoing posterior spinal fusion after lengthening were classified as the final fusion group (n = 9), while those with more than 12 months of follow-up without fusion formed the graduated group (n = 10). Demographics, surgical variables, and radiographic parameters (Cobb angle, correction rate, coronal balance, clavicular angle, thoracic kyphosis, lumbar lordosis, sagittal vertical axis) were compared. **Results**: Baseline characteristics were similar. At final follow-up, the final fusion group had significantly better outcomes in Cobb angle (24.2° vs. 34.9°, *p* = 0.002), correction rate (66.6% vs. 40.1%, *p* = 0.001), and coronal balance (−1.5 mm vs. 19.7 mm, *p* = 0.004). Sagittal alignment did not differ significantly, but preservation of thoracic kyphosis tended to favor the fusion group. **Conclusions**: Final fusion surgery after growing rod treatment achieved superior coronal correction and balance compared with observation alone. Although sagittal alignment was not statistically different, a trend toward better thoracic kyphosis preservation was observed. Final fusion should be considered for larger residual curves or coronal imbalance, while observation may suffice in well-corrected cases.

## 1. Introduction

Early-onset scoliosis (EOS) is defined as a spinal curvature of ≥10° in the coronal plane occurring before the age of 10 years, irrespective of etiology [1]. Unlike adolescent scoliosis, EOS arises during a critical period of spinal and thoracic growth and may result in severe consequences, including rapid curve progression, thoracic insufficiency, and impaired pulmonary development [2]. During this critical period, lung growth depends on adequate thoracic cavity development, including its height, width, and depth, which are determined by the coordinated growth of the spine, ribs, and sternum [3]. Insufficient thoracic development can compromise pulmonary capacity and contribute to thoracic insufficiency syndrome, underscoring the close relationship between spinal growth, thoracic growth, and cardiopulmonary function [4]. Accordingly, the primary treatment goal in EOS is not only deformity correction but also preservation of spinal and thoracic growth [5,6].

Given the young age of affected patients, nonoperative management with serial casting or bracing is typically considered the first-line treatment. Surgical intervention is generally reserved for patients with progressive curves that fail to respond to conservative therapy [7,8]. However, conventional spinal fusion in this population may arrest growth at the fused segments and result in complications such as the crankshaft phenomenon, in which continued anterior spinal growth induces rotational deformity around a fused posterior segment [9].

To address these concerns, fusionless surgical techniques, most notably the growing rod system, have been developed [10,11,12,13]. This technique involves segmental fixation at the proximal and distal ends of the curve, with the intervening segments left unfused to allow for continued spinal growth. Deformity control is achieved through serial lengthening procedures (growing rod lengthening), which provide periodic distraction of the rods in conjunction with the child’s growth and curve progression. More recently, alternatives such as magnetically controlled growing rods (MCGRs) and the Shilla growth guidance system have been introduced, which allow continued spinal growth without the need for repeated surgical lengthening procedures [14,15]. Despite its advantages, the growing rod system has inherent limitations. Repeated surgical interventions may lead to spontaneous spinal fusion, even without formal fusion procedures [16]. Furthermore, because the technique is fundamentally distraction-based, concerns have been raised regarding its potential to negatively impact sagittal alignment, particularly reductions in thoracic kyphosis and lumbar lordosis, which may have long-term functional consequences [17].

In many cases, a final fusion surgery is performed after sufficient spinal growth is achieved. However, some patients develop spontaneous fusion or do not undergo final fusion despite completing growing rod treatment. The long-term radiographic outcomes of these patients remain incompletely understood.

The aim of this study was to compare coronal and sagittal radiographic parameters between two groups of patients with EOS who underwent growing rod treatment: one group that underwent final fusion surgery, and another group that did not undergo final fusion after completion of growing rod treatment. This comparison may provide insight into the structural consequences of continued growth without final fusion, particularly in the sagittal plane.

## 2. Materials and Methods

This retrospective study included patients who underwent growing rod insertion for EOS at a single institution between 2015 and 2019. Among these patients, those who underwent definitive deformity correction and spinal fusion were classified as the final fusion group, while patients who had not undergone final fusion surgery but had more than 12 months of follow-up after their last growing rod lengthening were classified as the graduated group. Patients with less than 12 months of follow-up after their final lengthening procedure were excluded from the study.

For all included patients, clinical and radiographic data were collected longitudinally from the preoperative period, through the index surgery and subsequent lengthening procedures, until final fusion or the last available follow-up. The follow-up endpoint was defined as the last recorded visit with the treating team for patients who underwent distraction surgery or, if applicable, final fusion surgery.

Preoperative data included diagnosis, curve type (Lenke classifications), and Risser stage. Postoperative data included the number of lengthening procedures and the interval between the initial and final surgeries.

All patients underwent standing whole spine posteroanterior and lateral radiographs prior to surgery. Subsequent posteroanterior and lateral radiographs were obtained before and after each lengthening procedure, as well as at the final follow-up. The following radiographic parameters were measured and compared between the preoperative and final follow-up timepoints: Cobb angle, correction rate, coronal balance, clavicular angle, thoracic kyphosis, lumbar lordosis, and sagittal vertical axis.

The data analysis was performed using IBM SPSS Statistics for Windows, version 20.0.0 (IBM Corp., Armonk, NY, USA). Student’s *t*-test was used for the analysis of continuous variables and chi-squared test for the analysis of categorical variables. *p*-values ≤ 0.05 were considered statistically significant.

## 3. Results

A total of 19 patients who underwent growing rod fixation for EOS more than 12 months prior were included in this study. The patients were divided into two groups: the Graduated group (n = 10) and the Final fusion group (n = 9). The Graduated group included 7 females and 3 males who had not undergone final fusion surgery but were followed for more than 12 months after their last growing rod lengthening procedure. Among the 10 patients in this group, 8 underwent follow-up CT imaging, and spontaneous fusion of the lamina or facet joints was observed in 6 of them. The Final fusion group included 7 females and 2 males who underwent instrumented posterior spinal fusion after completion of the growing rod treatment. Patient demographics and surgical variables are summarized in Table 1.

The average age at initial surgery was 10.8 ± 2.3 years in the graduated group and 11.6 ± 2.2 years in the final fusion group. The average age at the last lengthening surgery (graduated group) or final fusion (final fusion group) was 14.9 ± 1.9 years and 15.2 ± 1.9 years, respectively. The mean follow-up duration was 35.1 ± 32.8 months in the graduated group and 39.1 ± 7.7 months in the final fusion group, with no statistically significant difference (*p* = 0.720). Overall, 16 of the 19 patients (84.2%) achieved a minimum follow-up of 24 months. During the initial growing rod fixation, the mean number of fused segments was significantly lower in the graduated group (8.5 ± 5.9) compared to the final fusion group (13.1 ± 1.1, *p* = 0.038). The average number of lengthening procedures was 1.2 ± 0.6 in the graduated group and 1.4 ± 0.5 in the final fusion group (*p* = 0.376). The interval between the first and last surgery was 26 ± 8.9 months in the graduated group and 34 ± 11.9 months in the final fusion group (*p* = 0.115). Etiologically, the graduated group consisted of 5 patients (50%) with idiopathic scoliosis and 5 (50%) with neuromuscular scoliosis. The final fusion group included 6 patients (66.7%) with idiopathic scoliosis and 3 (33.3%) with neuromuscular scoliosis (*p* = 0.463).

Preoperative radiographic measurements are shown in Table 2. The mean preoperative Cobb angle of the major curve was 60.3 ± 8.9° in the graduated group and 73.0 ± 28.8° in the final fusion group (*p* = 0.202). The mean preoperative thoracic kyphosis was 31.7 ± 17.4° in the graduated group and 34.5 ± 17.4° in the final fusion group (*p* = 0.685). The mean preoperative lumbar lordosis (L1–S1) was 48.8 ± 11.7° in the graduated group and 53.9 ± 22.6° in the final fusion group (*p* = 0.480). The sagittal vertical axis (SVA) was 17.3 ± 25.4 mm in the graduated group and 13.9 ± 52.6 mm in the final fusion group (*p* = 0.856). The preoperative coronal balance was 7.0 ± 18.4 mm in the graduated group and −9.4 ± 35.9 mm in the final fusion group (*p* = 0.220). The mean preoperative clavicular angle was −9.1 ± 20.6° in the graduated group and −1.5 ± 5.6° in the final fusion group.

At the final follow-up, coronal parameters are summarized in Table 3. The mean Cobb angle was 34.9 ± 5.3° in the graduated group (correction rate: 40.1 ± 15.8%) and 24.2 ± 11.9° in the final fusion group (correction rate: 66.6 ± 11.0%, *p* = 0.001). At the final follow-up, coronal balance increased to 19.7 ± 14.9 mm in the graduated group but improved to −1.5 ± 12.9 mm in the final fusion group (*p* = 0.004). The mean postoperative clavicular angle did not differ significantly between groups.

In terms of sagittal alignment, postoperative sagittal parameters are shown in Table 4. Thoracic kyphosis did not differ significantly between the graduated group (28.4 ± 12.9°) and the final fusion group (34.6 ± 10.6°, *p* = 0.268). When considering the change from baseline to the last follow-up, thoracic kyphosis decreased by −11.9 ± 16.4° in the graduated group, whereas it remained essentially unchanged in the final fusion group (0.1 ± 11.9°, *p* = 0.088). Lumbar lordosis decreased to 43.5 ± 15.2° in the graduated group and 49.0 ± 11.7° in the final fusion group (*p* = 0.399), with a mean change of −4.3 ± 12.9° and −4.9 ± 19.8°, respectively (*p* = 0.942). The SVA was −9.1 ± 42.8 mm in the graduated group and 1.1 ± 41.9 mm in the final fusion group (*p* = 0.604).

Overall, there were no significant differences in preoperative radiographic parameters between the two groups. However, the final fusion group demonstrated significantly superior correction in Cobb angle, correction rate, and coronal balance on final radiographic evaluation.

## 4. Discussion

The use of growing rod fixation for the treatment of EOS has become increasingly popular due to its ability to promote spine growth, correct deformity, and prevent further curve progression. Non-fusion treatment with growing rods is considered the gold standard for surgical management of EOS. It is recommended to undergo distraction every 6 months after growing rod fixation to stimulate spinal growth. However, the optimal time interval between distractions is still a matter of debate. Multiple lengthening operations may carry various complications, including those related to anesthesia, infection, psycho-social and economic factors, and spontaneous fusion due to prolonged immobilization [16,18]. When creating the final treatment strategy for EOS, it is important to consider comorbidities, repeated lengthening surgery and complications, as well as the expectations of each patient and their family members. This can make surgical treatment decisions more difficult.

After undergoing any type of fusionless surgery for EOS, the final step of the procedure involves the final instrumented fusion of the spine [19]. The final fusion of instrumentation usually involves removing previous implants, followed by re-instrumentation with further correction of the curve and fusion. Unless there has been a progression of the curve above, below, or both, the levels are typically the same as in the first surgical procedure. Balsano et al. recommend immediate final fusion surgery after the final distraction and skeletal maturity of the patient [20]. Through final fusion surgery, it is possible to achieve correction of residual deformity after lengthening and ensure stability of the instrumentation through solid spinal fusion.

In our study, the final Cobb angle was 24.2° in the final fusion group and 34.9° in the graduated group, corresponding to a correction rate of 66.6% and 40.1%, respectively. This indicates a significantly greater deformity correction in the final fusion group. While the average number of lengthening procedures was relatively low in both groups (1.4 and 1.2, respectively), this result suggests that final fusion can contribute to additional correction of residual deformity beyond the lengthening phase. When compared to previous studies, these correction rates are consistent with prior reports. Cahill et al. reported a total correction rate of 61% over the complete duration of management with growing rod treatment followed by final fusion surgery [21]. Similarly, our study confirms that main curve correction was achieved through final fusion, in line with previous reports. In contrast, Jain et al. reported lower correction rates of 37.9% in the final fusion group and 51.8% in the graduation group, despite a significantly greater number of lengthening procedures (mean 5.7 and 5.4, respectively) over a longer treatment duration (8.9 and 5.7 years) [22]. The greater correction observed in our final fusion group may be attributable to the use of osteotomies during the final fusion procedure, whereas the relatively limited correction in the graduated group may be related to the lower number of distractions and the absence of additional corrective surgery.

In addition, coronal balance was more favorable in the final fusion group compared to the graduated group. Although there were no statistically significant differences in sagittal vertical axis or clavicular angle, the final fusion group demonstrated overall superior deformity correction. In contrast, thoracic kyphosis and lumbar lordosis did not differ significantly between the two groups. However, when examining the change in thoracic kyphosis from the initial evaluation to the final follow-up, the final fusion group showed minimal change, decreasing only from 34.5° at baseline to 34.6° at the last follow-up with a mean difference of 0.1°, whereas the graduated group exhibited a noticeable decrease from 31.7° to 28.4°, corresponding to a mean difference of −11.9°. This suggests that the distraction-based correction method may contribute to a reduction in thoracic kyphosis over time. One possible explanation for the lack of a significant increase in thoracic kyphosis and lumbar lordosis in the final fusion group is that, unlike global sagittal parameters, these regional spinal curvatures vary widely among individuals and fall within a broad normal range [23,24,25]. Furthermore, although final fusion surgery may promote restoration of thoracic kyphosis and lumbar lordosis, the final measurements may have been underestimated due to the reduction in these curves during prior serial growing rod lengthening procedures.

**Figure 1:**
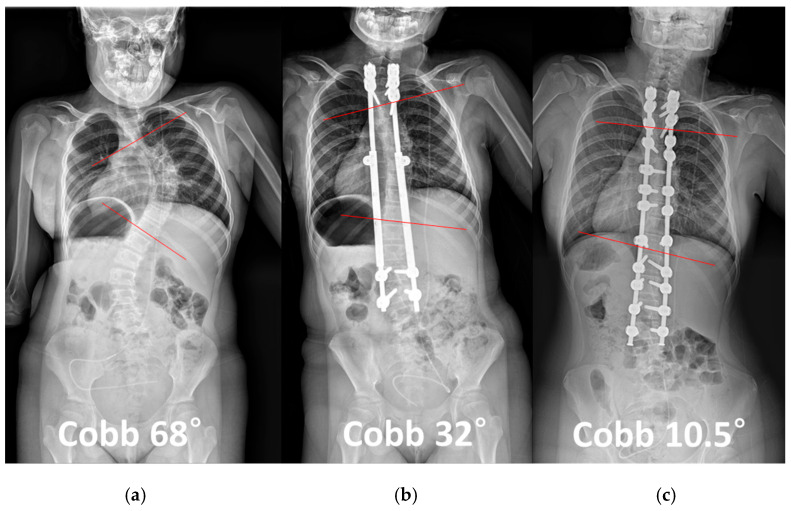
A 15-year-old female patient initially underwent growing rod insertion at the age of 8 for early-onset scoliosis. (**a**) Preoperative whole spine radiograph showing a major curve with a Cobb angle of 68°. (**b**) After growing rod lengthening, the Cobb angle improved to 32°. (**c**) Following final fusion surgery, further correction was achieved, with a Cobb angle of 10.5°. Red lines indicate the end vertebrae used to measure the Cobb angle. This case illustrates the substantial coronal plane correction provided by final fusion after growing rod treatment.

**Figure 2:**
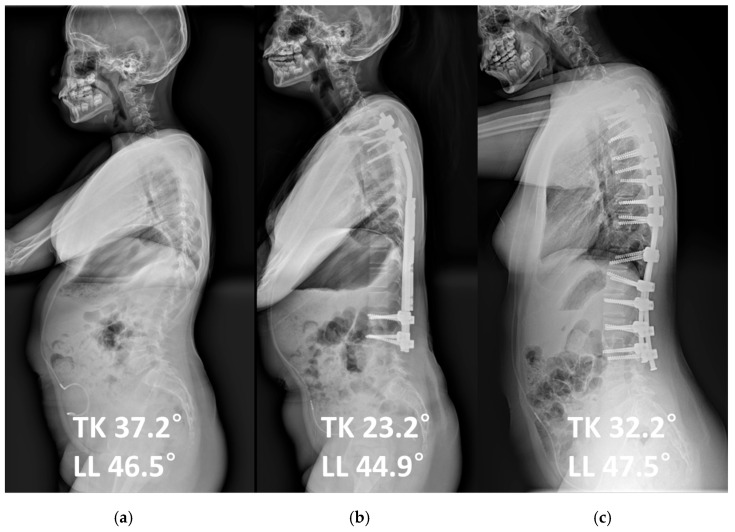
The same patient as in Figure 1 is shown in lateral radiographs. (**a**) Preoperative sagittal parameters included thoracic kyphosis (TK) of 37.2° and lumbar lordosis (LL) of 46.5°. (**b**) After growing rod lengthening, TK decreased to 23.2° and LL to 44.9°. (**c**) Following final fusion surgery, sagittal alignment improved with TK restored to 32.2° and LL to 47.5°. This case highlights the potential of final fusion to restore sagittal alignment, particularly thoracic kyphosis, after distraction-based correction.

As an illustrative case (Figure 1 and Figure 2), a 15-year-old female who initially underwent growing rod insertion at the age of 8 demonstrated marked coronal correction. Her major curve improved from 68° preoperatively to 32° after growing rod lengthening, and further decreased to 10.5° following final fusion surgery. In the sagittal plane, thoracic kyphosis decreased from 37.2° to 23.2° after growing rod treatment, but partially recovered to 32.2° after final fusion. Lumbar lordosis remained relatively stable, changing from 46.5° to 44.9° after lengthening and to 47.5° after final fusion. This case exemplifies how final fusion surgery may provide not only additional coronal correction but also partial restoration of thoracic kyphosis after distraction-based treatment.

Final fusion surgery can be expected to achieve deformity correction, as demonstrated in this study. However, the decision to perform final spinal fusion after growing rod treatment is complex and multifactorial. According to Flynn et al., only 15% of patients who underwent final fusion surgery after completing the final lengthening procedure had a correction of more than 50% due to stiffness [26]. Other studies have also found similar results regarding severe rigidity, spontaneous bony fusion, and limited deformity reduction [16,27]. Pizones et al. found that 93% of patients required multiple osteotomies to achieve corrections [28]. It is important that there is a high rate of complications and revision surgical rates following final instrumented fusion. Several authors have reported revision rates of 20–24% after final fusion surgery [29,30]. In particular, when spontaneous fusion has occurred, the necessity of performing surgery has been called into question.

Spontaneous fusion refers to unintended bony bridging at spinal levels not initially targeted for fusion, often observed in skeletally immature patients undergoing growing rod treatment. This phenomenon can impede further spinal growth and deformity correction. Its pathogenesis is thought to involve osteoprogenitor activity in damaged paraspinal muscle tissue, similar to the mechanism of heterotopic ossification seen after hip surgery. Reported rates of spontaneous fusion after growing rod fixation range from 81% to 89%, with risk factors including prolonged immobilization, muscle injury during exposure, immature vertebrae, and longer intervals between lengthening procedures [21,26]. Cahill et al. found that a longer interval of 10.4 months between distractions increased the risk of fusion, whereas more frequent lengthening procedures may mitigate it [21]. Over time, repeated lengthening procedures are also associated with increased spinal stiffness and reduced distraction efficacy [16,31]. Although some length gain may still occur post-spontaneous fusion due to bone remodeling, the rate is significantly diminished. Given that spontaneous fusion may reduce flexibility and limit additional correction, and that final fusion itself carries notable risks, the procedure may be avoidable in patients who have already reached skeletal maturity and acceptable alignment.

Therefore, when considering final fusion surgery after growing rod lengthening in patients with EOS, it is important to carefully assess radiographic parameters and the presence of spontaneous fusion. In particular, patients with a large residual Cobb angle or coronal imbalance after lengthening may benefit from final fusion surgery to achieve satisfactory outcomes. Additionally, in cases where spontaneous fusion is present, surgeons should be aware of potential stiffness and consider the possible need for osteotomies during final fusion. Conversely, if the patient demonstrates good coronal balance and adequate curve correction following lengthening, continued observation without final fusion may be a reasonable option. In such cases, when deformity correction is satisfactory and the patient is content with the outcome, final fusion surgery may not be strictly necessary. However, careful follow-up is essential to monitor potential implant-related complications such as loosening or breakage. Moreover, although not statistically significant in our series, final fusion may provide additional benefits in restoring thoracic kyphosis. For patients with markedly reduced kyphosis, an additional corrective procedure may still be advisable. Given the lack of long-term data extending into adulthood, further studies are warranted to determine the clinical implications of reduced sagittal alignment after EOS treatment. This study has several limitations. First, its retrospective design and relatively small sample size may limit the generalizability of the findings. Second, the number of lengthening procedures in both groups was lower than that reported in previous studies, which may have affected the correction rates and incidence of spontaneous fusion. Third, patient selection for final fusion versus observation was not randomized and may have been influenced by surgeon preference, patient comorbidities, or other unmeasured confounding factors. Finally, the absence of functional outcomes or patient-reported measures limits the ability to evaluate clinical benefits beyond radiographic correction. Future prospective studies with larger cohorts and longer follow-up are needed to validate these findings and guide surgical decision-making in EOS.

## 5. Conclusions

Final fusion surgery after growing rod treatment demonstrated superior radiographic outcomes compared with observation without final fusion, particularly in terms of Cobb angle correction, correction rate, and coronal balance. While sagittal alignment parameters did not reach statistical significance, the trend toward better preservation of thoracic kyphosis suggests potential benefit of final fusion. Based on these findings, final fusion surgery may be recommended for patients with larger residual curves or coronal imbalance after growing rod lengthening, whereas continued observation may be reasonable in cases with satisfactory correction and balance.

## Figures and Tables

**Table 1:** Patient demographics and surgical characteristics.

Variable	Graduated Group	Final Fusion Group	*p*-Value
Sex (female/male)	7/3	7/2	1.000
Age (Initial surgery) (years)	10.8 ± 2.3	11.6 ± 2.2	0.423
Age (Final surgery) (years)	14.8 ± 1.9	15.2 ± 1.9	0.640
Follow-up period (months)	35.1 ± 32.8	39.1 ± 7.7	0.720
Scoliosis type (Idiopathic/Congenital/ Neuromuscular/Syndromic)	5/0/5/0	6/0/3/0	0.463
Lenke classification (1/2/3/4/5/6)	5/0/2/1/0/2	1/1/6/0/0/1	0.138
Risser stage (0/1/2/3/4/5)	10/0/0/0/0/0	7/1/0/0/1/0	0.289
Fusion level	8.5 ± 5.9	13.1 ± 1.1	0.038 *
Number of lengthening	1.2 ± 0.6	1.4 ± 0.5	0.376
Time from initial to final surgery (months)	26.0 ± 8.9	34.0 ± 11.9	0.115

* Indicates statistical significance (*p*-value ≤ 0.05).

**Table 2:** Preoperative radiographic parameters.

Variable	Graduated Group	Final Fusion Group	*p*-Value
Cobb angle (main curve) (°)	60.3 ±8.9	73.0 ± 28.8	0.202
Coronal balance (mm)	7.0 ± 18.4	−9.4 ± 35.9	0.220
Clavicular angle (°)	−9.1 ± 20.6	−1.5 ± 5.6	0.306
Thoracic kyphosis (°)	31.7 ± 17.4	34.5 ± 17.4	0.685
Lumbar lordosis (°)	48.8 ± 11.7	53.9 ± 22.6	0.480
Sagittal vertical axis (mm)	17.3 ± 25.4	13.9 ± 52.6	0.856

**Table 3:** Postoperative coronal radiographic outcomes.

Variable	Graduated Group	Final Fusion Group	*p*-Value
Cobb angle (main curve) (°)	34.9 ± 5.3	24.2 ± 11.9	0.020 *
Correction rate of main curve (%)	40.1 ± 15.8	66.6 ± 11.0	0.001 *
Coronal balance (mm)	19.7 ± 14.9	−1.5 ± 12.9	0.004 *
Clavicular angle (°)	8.7 ± 22.3	2.6 ± 5.4	0.434

* Indicates statistical significance (*p*-value ≤ 0.05).

**Table 4:** Postoperative sagittal radiographic outcomes.

Variable	Graduated Group	Final Fusion Group	*p*-Value
Sagittal vertical axis (mm)	−9.1 ± 42.8	1.15 ± 41.9	0.604
Thoracic kyphosis (°)	28.4 ± 12.9	34.6 ± 10.6	0.268
Lumbar lordosis (°)	43.5 ± 15.2	49.0 ± 11.7	0.399
Δ Thoracic kyphosis (°)	−11.9 ± 16.4	0.1 ± 11.9	0.088
Δ Lumbar lordosis (°)	−4.3 ± 12.9	−4.9 ± 19.8	0.942

Δ indicates the change from baseline to final follow-up.

## Data Availability

The data underlying this article cannot be shared publicly because of the privacy of the individuals who participated in this study. The data can be shared by the corresponding authors upon reasonable request.
